# Utilization of glycated hemoglobin testing in the Korean health check-up population from local clinics and hospitals and its impact on diabetes and prediabetes classification

**DOI:** 10.1186/s12902-026-02289-9

**Published:** 2026-05-12

**Authors:** Rihwa Choi, Mi-Jung Park, Sang Gon Lee

**Affiliations:** 1 Laboratory Medicine Center, GC Labs, 107, Ihyeonro 30 beon-gil, Giheng-gu, Yongin-Si, Gyeonggi-do 16924 Korea; 2 Department of Laboratory Medicine and Genetics, Sungkyunkwan University School of Medicine, 81 Irwon-ro, Gangnam-gu, Seoul, 06351 Korea

**Keywords:** Diabetes, Prediabetes, Glucose, Hemoglobin A1c, Utilization, Cutoff

## Abstract

**Background:**

Monitoring test utilization is a key component of improving the quality of clinical laboratory testing. In diabetes management, glycated hemoglobin (hemoglobin A1c, HbA1c) reflects long-term glycemic control in addition to blood glucose levels; however, the combined use of these tests in routine health checkups has not been well evaluated in Korea.

**Methods:**

We retrospectively analyzed data from routine health checkups conducted between 2018 and 2023 that included concurrent serum glucose and HbA1c measurements. Diabetes and prediabetes were defined using glucose-only, HbA1c-only, and combined (glucose or HbA1c) diagnostic criteria. Prevalence estimates across diagnostic definitions were compared, and factors associated with diabetes and prediabetes were evaluated using multivariable logistic regression adjusted for sex, age, and year of testing.

**Results:**

Over the 6-year study period, test results from 113,790 individuals (56,021 men and 57,769 women) were analyzed, with a mean age of 55.4 years (SD, 13.3). The number of individuals undergoing simultaneous testing increased from 5,922 in 2018 to 36,814 in 2023, indicating increased utilization of HbA1c testing. The combined diagnostic criteria identified significantly more individuals with diabetes and prediabetes than either glucose-only or HbA1c-only definitions (*p* < 0.001). In multivariable analyses, male sex and older age were independently associated with higher odds of diabetes, whereas the prevalence of prediabetes increased in more recent years of testing.

**Conclusions:**

Simultaneous evaluation using serum glucose and HbA1c significantly improves the detection of diabetes and prediabetes compared with single-marker strategies. These findings underscore the importance of optimized test utilization in routine health checkups to enhance the early identification of dysglycemia.

**Clinical trial number:**

Not applicable.

## Introduction

In diabetes screening and management, blood glucose and glycated hemoglobin (hemoglobin A1c, HbA1c) represent complementary biomarkers: blood glucose reflects short-term glycemic status, whereas HbA1c provides an integrated measure of long-term glycemic control [1–3]. Reliance on a single biomarker may therefore result in missed or delayed identification of dysglycemia [4–6].

In Korea, adults undergo a National Health Insurance Service (NHIS)–supported general health examination, in which fasting blood glucose is currently the only diabetes-related laboratory test included [6, 7]. Although HbA1c is widely recognized as an essential marker for diabetes management, it has not yet been incorporated into the national health screening program [1–7]. The Committee on Health Insurance and Government Relations of the Korean Diabetes Association has issued a position statement emphasizing the clinical appropriateness and public health significance of adding HbA1c to the national health examination, noting that combined testing could identify a greater number of individuals eligible for timely intervention [6]. However, real-world evidence evaluating the impact of combined glucose and HbA1c testing in routine health checkup settings in Korea remains limited [4–6, 8–10].

From a laboratory medicine perspective, appropriate test utilization is a fundamental component of clinical laboratory quality, as it directly influences diagnostic accuracy, clinical decision-making, and population-level disease detection [11–15]. Furthermore, utilization analysis provides objective data to guide test stewardship initiatives, including the development of reflex or combined testing strategies that improve diagnostic yield while maintaining cost-effectiveness [11–17].

Accordingly, this study aimed to evaluate trends in HbA1c test utilization using real-world laboratory data from individuals who underwent routine health checkups at local clinics and hospitals with concurrent serum glucose and HbA1c measurements. In addition, we sought to assess differences in the proportions of individuals classified as having diabetes or prediabetes when glycemic status was defined using glucose-only, HbA1c-only, or combined glucose and HbA1c criteria.

## Methods

### Study population and data collection

We conducted a retrospective, laboratory-based study using health checkup data obtained from GC Labs, Korea. Adults who visited local clinics and hospitals and underwent both serum glucose and HbA1c testing as part of routine health checkups between January 1, 2018, and December 31, 2023, were eligible for inclusion. Individuals were instructed to fast for at least 8 h before blood collection in accordance with routine health checkup protocols [3, 7]. To avoid duplication, repeated measurements from the same individual were excluded, and only the first available test result per individual was included in the analysis. Individuals with missing serum glucose or HbA1c results were excluded from analyses requiring paired comparisons. Age was categorized into predefined 10-year intervals (20–29, 30–39, 40–49, 50–59, 60–69, 70–79, and ≥ 80 years).

### Laboratory measurements

Serum glucose concentrations were measured using automated cobas 8000 c702 analyzers (Roche Diagnostics, Mannheim, Germany). HbA1c levels were measured using the Tina-quant Hemoglobin A1c Gen.3 reagent on cobas c513 analyzers (Roche Diagnostics, Mannheim, Germany). Both assays were performed using standardized and traceable analytical methods, and the measurement platforms and methodologies remained unchanged throughout the study period [18, 19].

### Definitions of diabetes and prediabetes

Diabetes and prediabetes were classified using three diagnostic approaches: glucose-only, HbA1c-only, and combined criteria. Under the glucose-only definition, diabetes was defined as a serum glucose level ≥ 126 mg/dL, and prediabetes as a serum glucose level of 100–125 mg/dL [1–3]. Under the HbA1c-only definition, diabetes was defined as an HbA1c level ≥ 6.5%, and prediabetes as an HbA1c level of 5.7–6.4% [1–3]. Under the combined definition, diabetes was defined as meeting either the glucose or HbA1c criterion for diabetes, while prediabetes was defined as meeting either prediabetes criterion in the absence of diabetes [1–3, 20]. Individuals who did not meet any of these criteria were classified as having normal glycemic status.

### Statistical analysis

Descriptive analyses were conducted to estimate the prevalence of diabetes and prediabetes according to each diagnostic definition. Annual prevalence of diabetes and prediabetes was calculated by age group and sex under each diagnostic criterion. Prevalence estimates were expressed as percentages, using the number of individuals with available measurements as the denominator.

Multivariable logistic regression analyses were performed to identify factors associated with diabetes and prediabetes. Separate models were constructed for diabetes and for prediabetes, with individuals with diabetes excluded from the prediabetes model. Sex, age group, and year of testing were included as independent variables. Odds ratios (ORs) and 95% confidence intervals (CIs) were estimated, and results were visualized using forest plots with ORs displayed on a logarithmic scale.

To evaluate concordance and discordance between glucose-based and HbA1c-based classifications, Venn diagrams were constructed for diabetes and prediabetes based on the combined (“glucose or HbA1c”) definition. Analyses were performed for the overall study population and stratified by sex.

A p-value < 0.05 was considered statistically significant. All statistical analyses and visualizations were performed using R software (version 4.5.2; R Foundation for Statistical Computing, Vienna, Austria).

## Results

A retrospective review of GC Labs laboratory data identified 1,180,837 glucose tests performed for health checkup purposes at local clinics and hospitals between 2018 and 2023. Of these, 134,725 were ordered concurrently with HbA1c. After limiting the dataset to Korean adults aged ≥ 20 years, 134,609 cases remained. When repeated measurements were present, only the first concurrent glucose and HbA1c test result per individual was retained, yielding a final analytic cohort of 113,790 individuals. The final study population included 56,021 men and 57,769 women, with a mean age of 55.4 ± 13.3 years. Table 1 summarizes the demographic characteristics of the study population and the distribution of glycemic status according to different diagnostic definitions. The cohort predominantly consisted of middle-aged to older adults, with individuals aged 40–69 years accounting for approximately three-quarters of the population, reflecting the age structure of individuals undergoing routine health examinations.

**Table 1:** Characteristics of study subjects (*n* = 113,790)

Characteristics	Number of subjects	(%)	Glucose, mg/dL		HbA1c, %NGSP unit	
			Median	25th-75th	Median	25th-75th
Gender			97	88–113	5.9	5.6–6.4
Men	56,021	49.2	94	91–120	6.3	5.6–6.7
Women	57,769	50.8	101	87–107	6.1	5.5–6.3
Age group						
20 ~ 29 years	3,609	3.2	87	82–93	5.4	5.2–5.6
30 ~ 39 years	10,504	9.2	91	84–99	5.5	5.3–5.8
40 ~ 49 years	22,520	22.4	94	87–107	5.7	5.4–6.1
50 ~ 59 years	29,994	26.4	98	89–115	5.9	5.6–6.5
60 ~ 69 years	27,933	24.5	101	91–118	6.1	5.7–6.7
70 ~ 79 years	13,159	11.6	104	92–123	6.2	5.8–6.9
≥ 80 years	3,071	2.7	104	93–123	6.2	5.8–6.8
Year of testing						
2018	5,922	5.2	110	95–134	6.2	5.6–7.1
2019	7,798	6.8	106	93–129	6.1	5.6–7.0
2020	11,399	10.0	102	91–123	5.9	5.5–6.7
2021	19,567	17.2	97	88–113	5.9	5.6–6.5
2022	32,320	28.4	96	88–110	5.8	5.5–6.3
2023	36,814	32.4	95	87–108	5.8	5.6–6.3
Glucose-only definition						
Normal	62,771	55.2	90	84–95	5.6	5.4–5.9
Prediabetes	32,732	28.8	109	104–116	6.2	5.8–6.6
Diabetes	18,287	16.1	148	135–176	7.4	6.8–8.6
HbA1c-only definition						
Normal	37,464	32.9	89	83–95	5.4	5.3–5.6
Prediabetes	47,894	42.1	97	89–106	5.9	5.8–6.1
Diabetes	28,432	25.0	129	113–154	7.2	6.7–8.0
Either the glucose or HbA1c						
Normal	32,056	28.2	88	82–93	5.4	5.3–5.5
Prediabetes	50,867	44.7	98	90–106	5.9	5.7–6.1
Diabetes	30,867	27.1	131	114–153	7.1	6.7–7.9

For the glucose-only definition, diabetes was defined as a serum glucose level ≥ 126 mg/dL and prediabetes as a serum glucose level of 100–125 mg/dL [1–3]. For the HbA1c-only definition, diabetes was defined as an HbA1c level ≥ 6.5%, and prediabetes as an HbA1c level of 5.7–6.4% [1–3]. For the combined definition, diabetes was defined as meeting either the glucose or HbA1c criteria for diabetes, and prediabetes was defined as meeting either prediabetes criterion in the absence of diabetes [1–3, 20]. Individuals not meeting any of these criteria were classified as normal

The number of individuals undergoing simultaneous serum glucose and HbA1c testing increased markedly from 5,922 in 2018 to 36,814 in 2023, indicating a substantial rise in HbA1c utilization in routine health checkups. The volume of concurrent testing increased steadily over time, particularly from 2021 onward, with more than 60% of all tests performed in 2022 and 2023. Marked differences were observed in the distribution of glycemic categories depending on the diagnostic criteria applied.

**Fig. 1:**
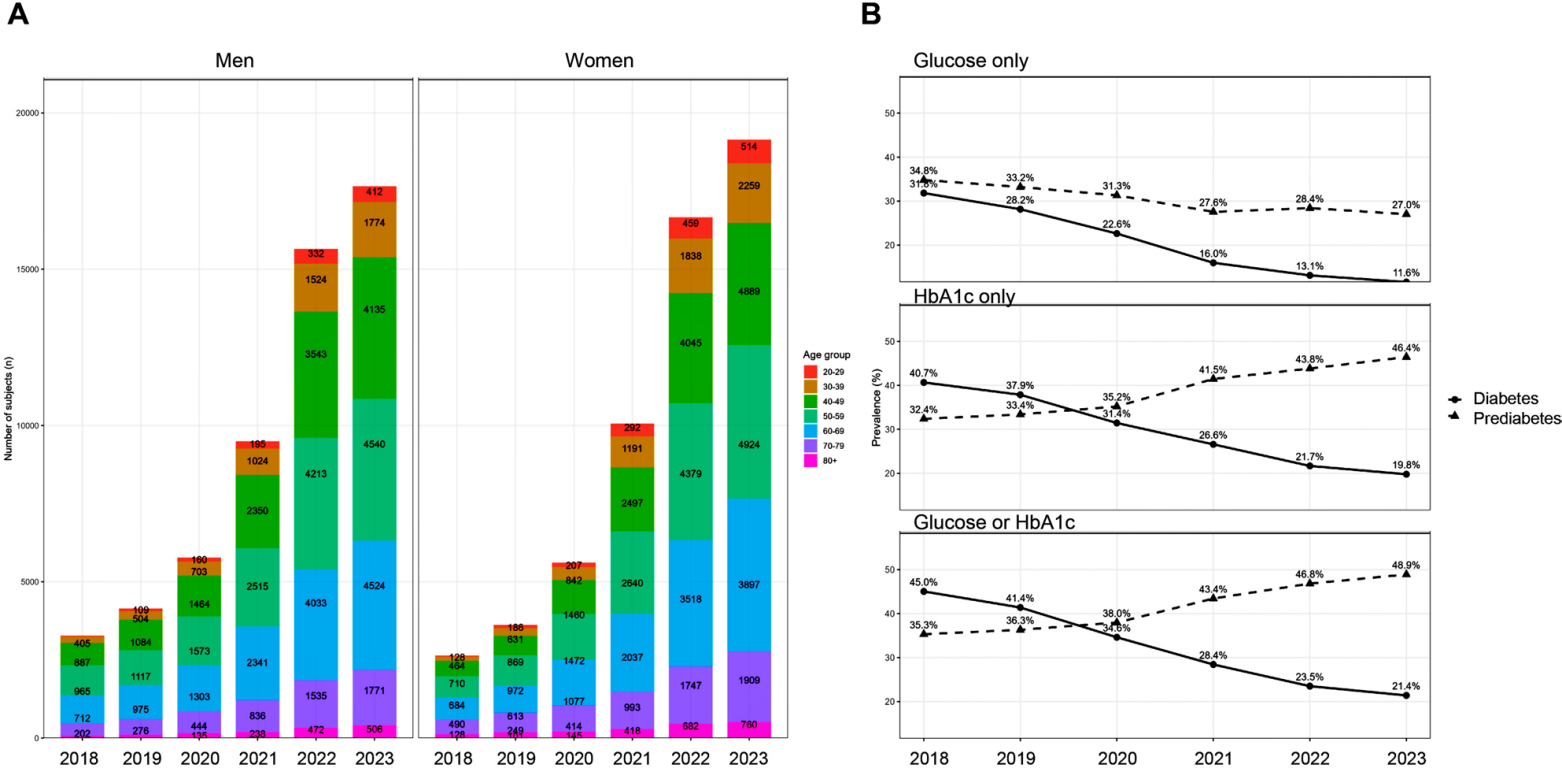
Annual number of individuals undergoing simultaneous serum glucose and HbA1c testing and the prevalence of diabetes and prediabetes. (**A**) Annual number of individuals stratified by age group and sex. (**B**) Annual prevalence of diabetes and prediabetes according to glucose-only, HbA1c-only, and combined (glucose or HbA1c) diagnostic criteria

Figure 1A illustrates the annual number of individuals tested, stratified by sex and age group, demonstrating that most participants were middle-aged or older adults, whereas individuals aged ≥ 80 years accounted for a relatively small proportion across all years. Figure 1B shows the annual prevalence of diabetes and prediabetes according to different diagnostic criteria. Prediabetes was consistently more prevalent than diabetes, and prevalence estimates were highest when combined glucose or HbA1c criteria were applied, although overall temporal trends were similar across definitions.

**Fig. 2:**
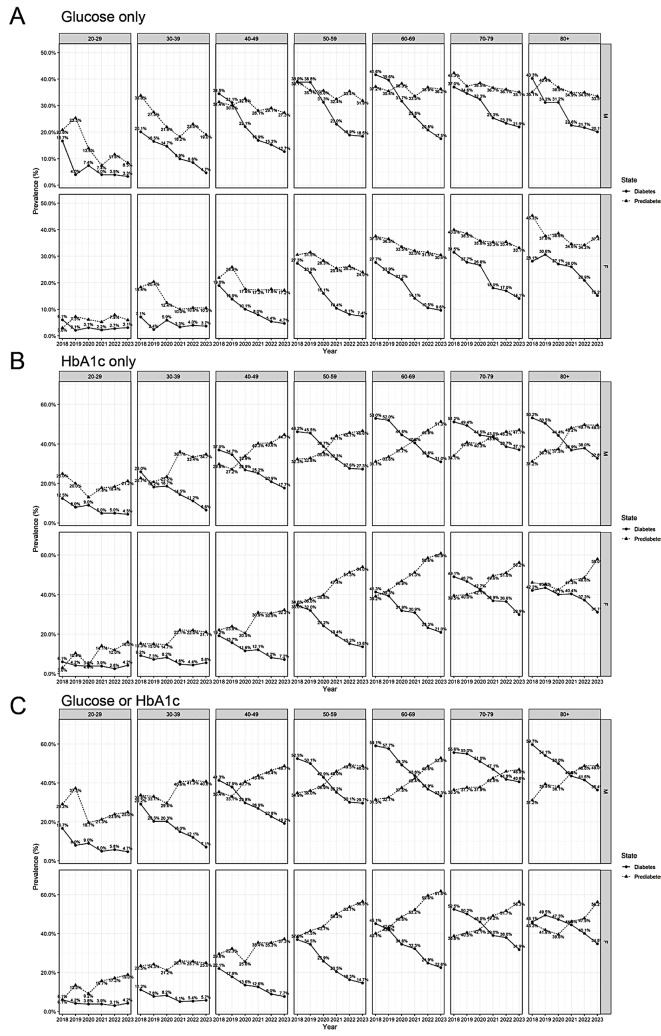
Annual prevalence of diabetes and prediabetes stratified by sex and age group according to diagnostic criteria. (**A**) Glucose-only criteria. (**B**) HbA1c-only criteria. (**C**) Combined (glucose or HbA1c) diagnostic criteria

The prevalence of both diabetes and prediabetes increased progressively with age and differed by sex across all diagnostic criteria (Fig. 2). Prevalence estimates based on serum glucose alone, HbA1c alone, and combined glucose or HbA1c criteria are presented separately for men and women. Across all age groups, the prevalence of diabetes and prediabetes was consistently higher in men than in women (*p* < 0.05).

**Fig. 3:**
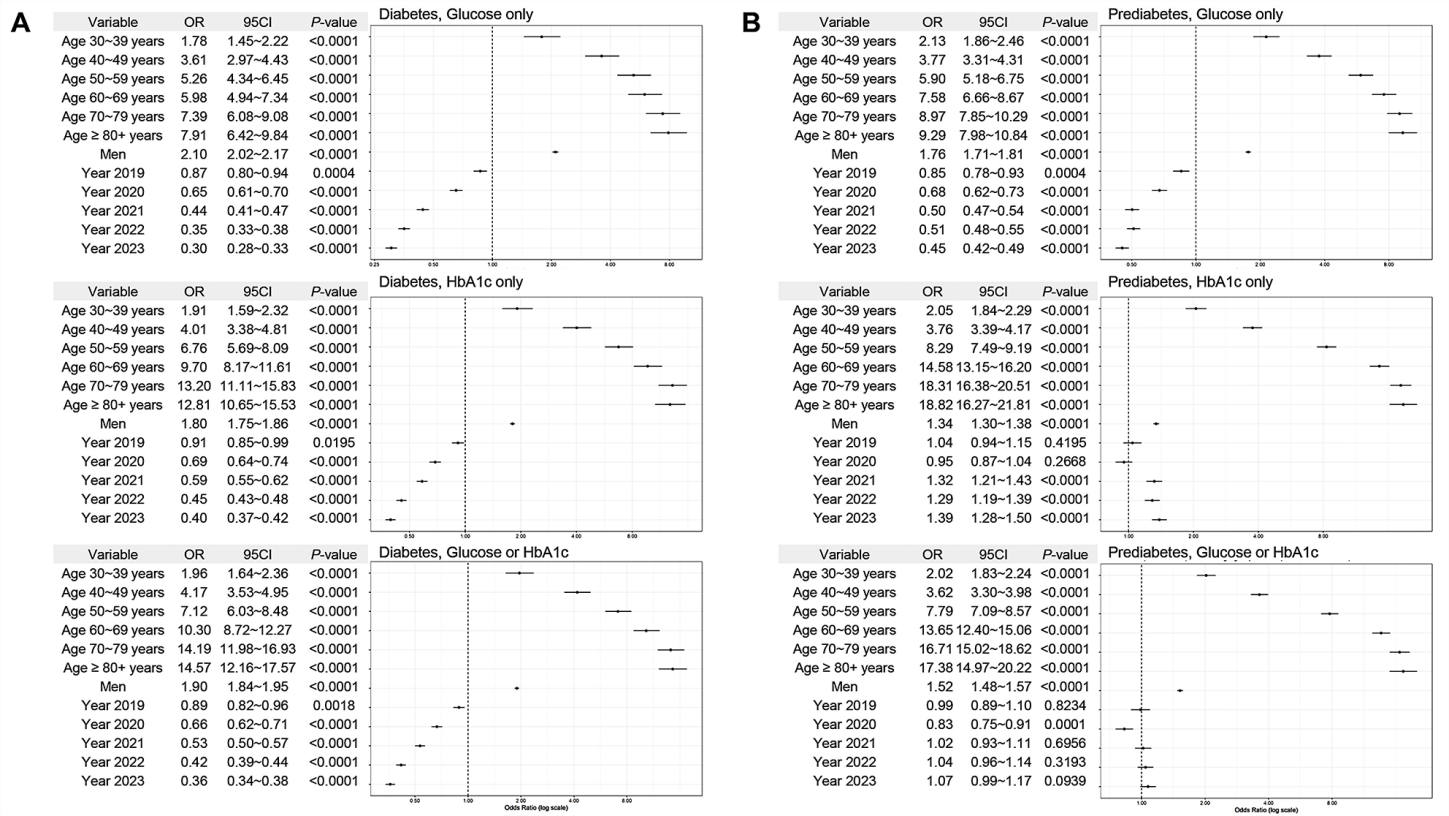
Forest plots of factors associated with diabetes (**A**) and prediabetes excluding diabetes (**B**) based on glucose only, HbA1c only, and glucose or HbA1c criteria. Odds ratios are presented on a logarithmic scale with 95% confidence intervals

Multivariable logistic regression analyses were conducted to identify factors independently associated with diabetes and prediabetes among health checkup participants. Increasing age was the strongest independent factor associated with both diabetes and prediabetes (Fig. 3), with progressively higher odds observed in older age groups after multivariable adjustment. Male sex was also independently associated with higher odds of diabetes and prediabetes. Calendar year of testing showed a significant inverse association with diabetes classification; compared with earlier years, later years were associated with lower adjusted odds of diabetes after accounting for age and sex. With respect to diagnostic criteria, HbA1c-based definitions (HbA1c-only or combined glucose or HbA1c) were associated with higher odds of diabetes detection compared with glucose-only criteria. However, the magnitude of this association was modest relative to that observed for prediabetes, suggesting that HbA1c contributed incrementally rather than substantially altering diabetes classification. Detailed results are presented as forest plots with odds ratios displayed on a logarithmic scale.

**Fig. 4:**
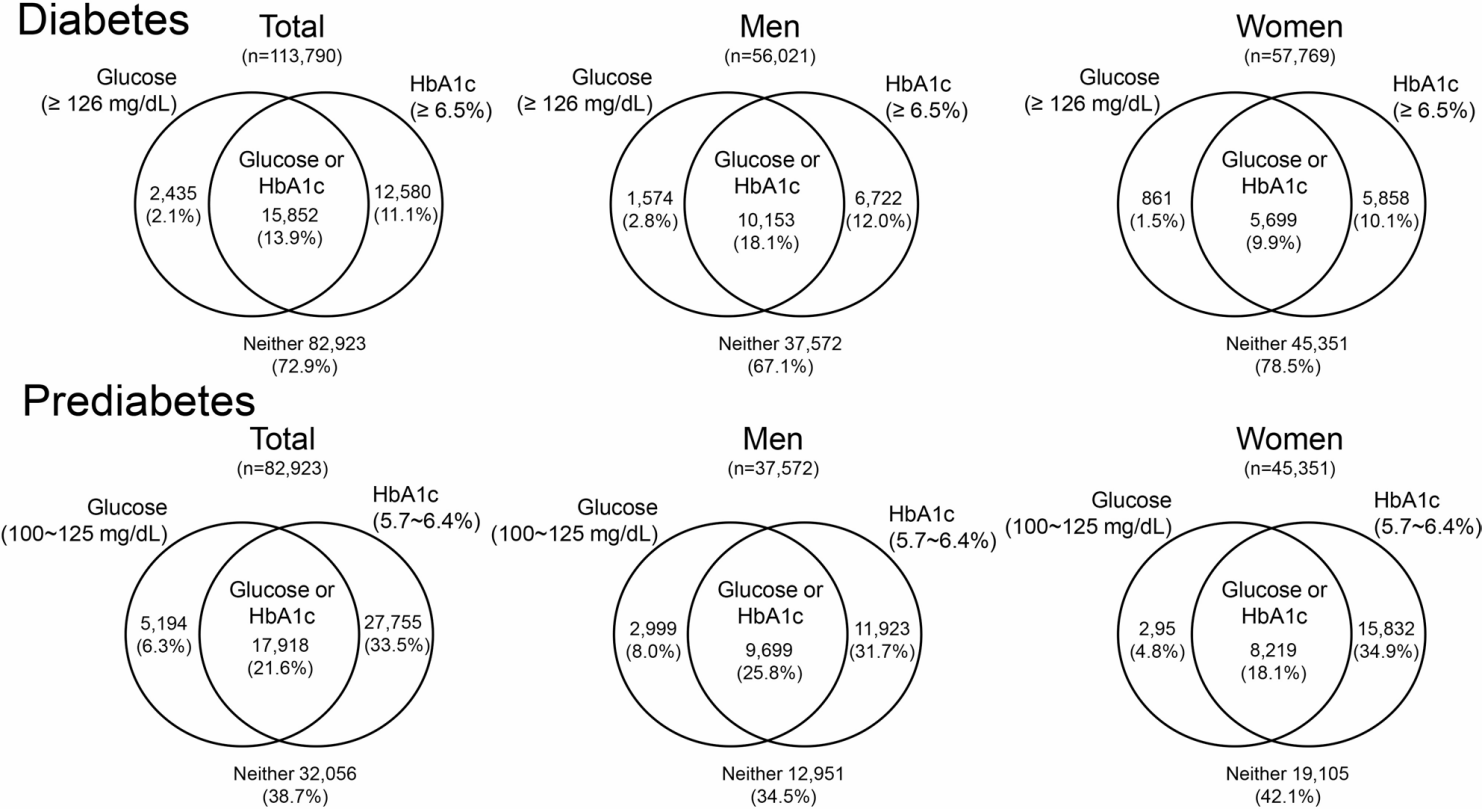
Venn diagrams illustrating the overlap between glucose-based and HbA1c-based classifications for diabetes and prediabetes by sex using the glucose or HbA1c definition. Diagrams display the number and percentage of subjects identified by glucose only, HbA1c only, both criteria, or neither

Simultaneous evaluation using combined criteria identified a higher proportion of individuals with diabetes and prediabetes than glucose-only testing (Fig. 4). When diabetes was defined using combined criteria (glucose or HbA1c), prevalence estimates were significantly higher than those based on glucose-only or HbA1c-only definitions (all *p* < 0.001). Using the combined definition, 30,867 of 113,790 individuals (27.1%) met the criteria for diabetes, and 50,867 (44.7%) met the criteria for prediabetes. Notably, HbA1c-based classification identified a substantial proportion of individuals who were not captured by glucose alone. Approximately one in ten individuals in the overall population was classified as having diabetes based solely on HbA1c values (12,580/113,790), and nearly one in four individuals was classified as having prediabetes based solely on HbA1c (27,755/113,790).

**Fig. 5:**
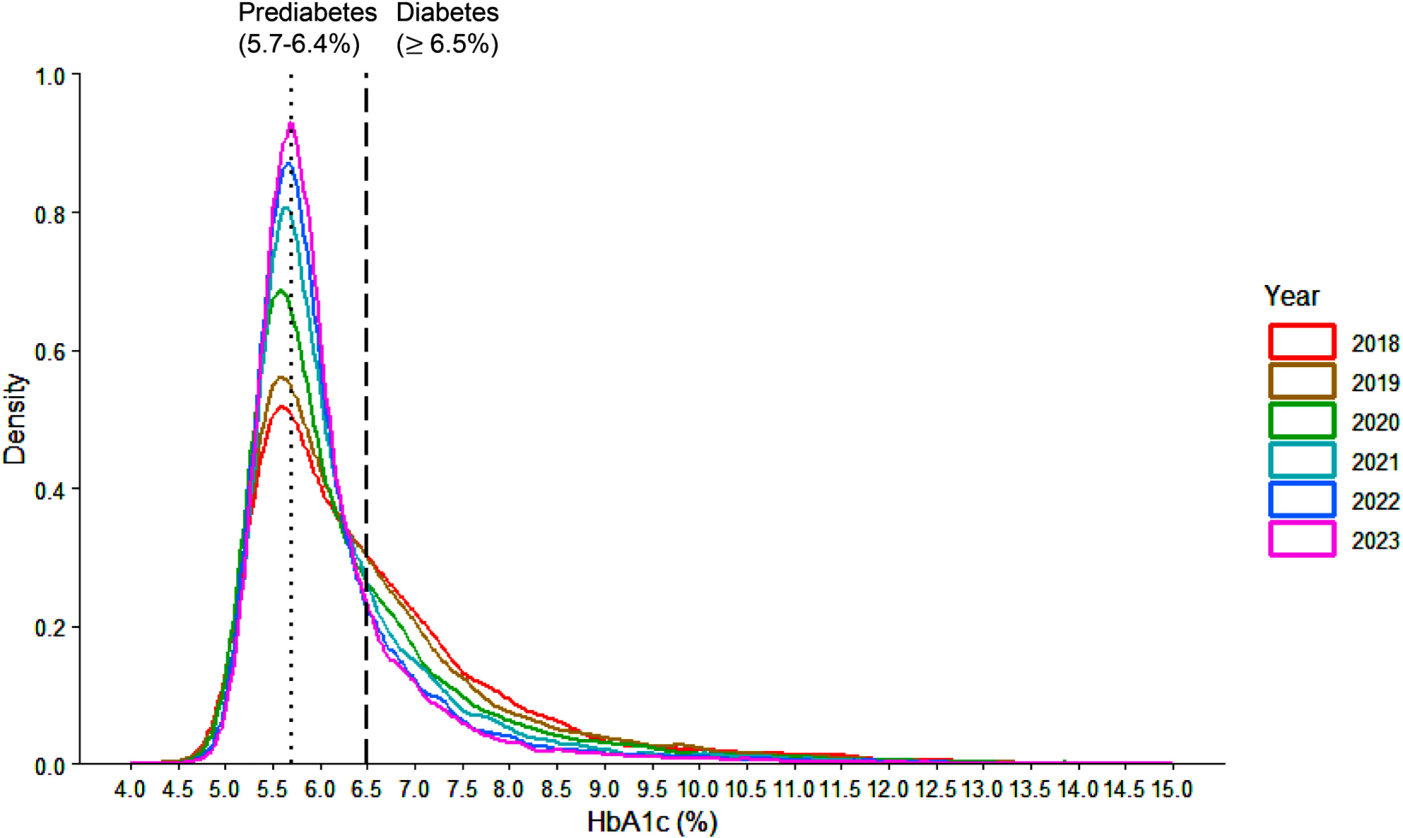
Annual distribution of HbA1c values shown as density plots by year. Each curve represents the kernel density estimate of HbA1c (%) for a given calendar year. Vertical reference lines indicate the diagnostic thresholds for prediabetes (HbA1c 5.7%; dotted line) and diabetes (HbA1c 6.5%; long-dashed line)

Figure 5 shows the annual distribution of HbA1c values. Although the overall shape of the distribution remained relatively stable across years, subtle shifts were observed around diagnostic cutoffs, particularly near the prediabetes threshold (5.7%), suggesting that small changes in HbA1c distribution may meaningfully influence prevalence estimates.

## Discussion

This laboratory-based study demonstrates a marked increase in the utilization of HbA1c testing during routine health checkups over a six-year period in Korea. This temporal pattern reflects a substantial expansion in testing volume in recent years and provides a robust basis for evaluating contemporary diagnostic practices. Over the study period, the prevalence of diabetes declined steadily, whereas the prevalence of prediabetes increased in both sexes. These findings suggest that temporal differences in prevalence estimates should be interpreted in the context of diagnostic definitions as well as shifts in the age and sex distribution of the screened population. Density analyses revealed increasing clustering of HbA1c values near the prediabetes cutoff, indicating that observed changes in prevalence largely reflect a growing proportion of individuals with borderline glycemic abnormalities. The concurrent decline in diabetes prevalence and rise in prediabetes prevalence may therefore reflect broader screening of non-diabetic individuals as HbA1c testing becomes more widely adopted, rather than a true reduction in disease burden [20–25]. Alternatively, improved disease awareness, treatment, and glycemic control among individuals with established diabetes may have resulted in fewer diabetic individuals presenting in routine screening settings [20–25]. The concurrent ordering of HbA1c in addition to fasting serum glucose may also reflect physicians’ clinical judgment that certain individuals were at increased risk of dysglycemia and required more comprehensive glycemic evaluation [1–3]. Consistent with this interpretation, the Diabetes Fact Sheet in Korea 2024 reports ongoing improvements in diabetes awareness, treatment, and control rates [20, 21]. Because this study was based on data from a single large commercial laboratory, the observed increase in concurrent HbA1c testing should be interpreted cautiously, as it may reflect changes in physician ordering practices, GC Labs’ service expansion, or changes in health check-up panel contracts over time. In addition, because HbA1c is not currently included in the NHIS national health screening program, external benchmark data are not available, making it difficult to determine whether this trend reflects broader changes in market share or nationwide screening practice. In addition, the study period also overlapped with the COVID-19 pandemic, which substantially disrupted healthcare utilization and routine screening in Korea [15]. Therefore, the marked increase in testing volume observed in 2021–2022 may partly reflect the resumption of delayed health checkups after pandemic-related disruptions, which may also have influenced the observed temporal patterns of dysglycemia prevalence [15].

Simultaneous application of serum glucose and HbA1c substantially enhanced the detection of both diabetes and prediabetes compared with glucose testing alone in the present study. These findings highlight the complementary diagnostic contribution of HbA1c in identifying dysglycemia in a large health-screening population [4–6, 9, 22–25]. The substantial discordance observed between glucose- and HbA1c-based classifications underscores the limitations of single-marker approaches and supports the value of combined testing strategies for early detection of dysglycemia [4–6, 9]. Population-based health examination surveys and studies from global consortia have reported age-standardized diabetes prevalences of approximately 12%, demonstrating considerable heterogeneity in the proportions of individuals with isolated elevated fasting glucose, isolated elevated HbA1c, or elevations in both markers according to ethnicity, age, and country income level [5]. In these studies, among individuals with screen-detected diabetes, approximately 29% had isolated elevated fasting plasma glucose, 37% had isolated elevated HbA1c, and 34% had elevations in both glucose and HbA1c [5].

According to the Diabetes Fact Sheet in Korea 2024, which analyzed KNHANES 2021–2022 data, the prevalence of diabetes and prediabetes among Korean adults aged ≥ 30 years was 14.8% and 41.1%, respectively. Notably, diabetes prevalence increased from 14.2% based on fasting plasma glucose alone to 15.5% when HbA1c and a history of physician-diagnosed diabetes or treatment were incorporated into the definition [20]. In the present study, the addition of HbA1c to glucose-based criteria resulted in higher diabetes prevalence estimates, with increases of approximately 1.5% in women and 2.8% in men, findings that are comparable to those reported in the national statistics [20, 21]. In contrast, the incremental impact of HbA1c was substantially greater for prediabetes, with additional prevalence estimates of 11.9% in men and 19.8% in women. Increasing attention has therefore been directed toward improving awareness and detection of prediabetes, as early identification and intervention before progression to overt diabetes are expected to yield substantial clinical and public health benefits [22–26]. While much of the existing literature has focused on diabetes, recent studies have increasingly examined the cost-effectiveness and clinical utility of combined glucose and HbA1c testing for the detection of prediabetes [22–32].

Several limitations should be acknowledged. This study was retrospective and relied on laboratory test data without detailed clinical information, which may have resulted in misclassification of glycemic status. The absence of information on previously diagnosed diabetes and antidiabetic treatment may have led to inclusion of individuals with established disease, potentially affecting both prevalence estimates and the perceived added value of combined HbA1c and glucose testing. Fasting status could not be fully verified, and the health checkup population may not be representative of the general Korean population. Although fasting status could not be individually verified in this retrospective dataset, routine health checkups at local clinics and hospitals in Korea are generally performed under standardized protocols requiring at least 8 h of fasting before blood collection [3, 7]. Therefore, while some misclassification due to non-fasting samples cannot be excluded, the reliability of glucose-based classification is likely preserved in most participants. Because HbA1c is not included in the mandatory NHIS general health examination, individuals undergoing both glucose and HbA1c testing may represent a selected subpopulation with higher baseline risk of dysglycemia, greater health awareness, or access to clinics that actively promote add-on testing. Therefore, the study cohort may differ from the general Korean screening population, which should be considered when interpreting the prevalence estimates and generalizability of our findings. In addition, only a single measurement per individual was analyzed, and conditions known to affect HbA1c levels could not be fully accounted for.

Despite these limitations, this real-world laboratory-based study demonstrates that combined glucose and HbA1c testing enhances the detection of dysglycemia and supports optimized test utilization in routine health checkups. From a laboratory medicine perspective, these findings underscore the clinical value of combined testing strategies and illustrate how test utilization patterns can influence epidemiologic estimates derived from screening populations [11–14].

## Conclusions

In conclusion, the utilization of HbA1c testing in routine health checkups at local clinics and hospitals in Korea has increased substantially over the past six years. Simultaneous measurement of serum glucose and HbA1c improves the detection of diabetes and prediabetes, supporting the role of HbA1c as a complementary screening tool in routine clinical practice. However, because a substantial proportion of individuals cluster near diagnostic cutoffs, particularly around the prediabetes threshold, prevalence estimates may be highly sensitive to small shifts in the underlying distribution of test values. This finding underscores the need for cautious interpretation of prevalence estimates derived from screening populations, especially when comparing results across time periods or diagnostic definitions. Continued monitoring of test utilization patterns, together with careful consideration of cutoff-related distributional effects, is warranted to optimize laboratory-driven strategies for diabetes and prediabetes screening.

## Data Availability

The datasets generated and analyzed during the current study are available from the corresponding authors on reasonable request.

## Abbreviations

NHIS: National Health Insurance Service
HbA1c: Hemoglobin A1c (glycated hemoglobin)
OR: Odds ratio
CI: Confidence interval
